# Designing peptides predicted to bind to the omicron variant better than ACE2 via computational protein design and molecular dynamics

**DOI:** 10.1371/journal.pone.0292589

**Published:** 2023-10-10

**Authors:** Thassanai Sitthiyotha, Wantanee Treewattanawong, Surasak Chunsrivirot

**Affiliations:** Structural and Computational Biology Research Unit, Department of Biochemistry, Faculty of Science, Chulalongkorn University, Pathumwan, Bangkok, Thailand; Nazarbayev University School of Medicine, PAKISTAN

## Abstract

Brought about by severe acute respiratory syndrome coronavirus 2 (SARS-CoV-2), coronavirus disease (COVID-19) pandemic has resulted in large numbers of worldwide deaths and cases. Several SARS-CoV-2 variants have evolved, and Omicron (B.1.1.529) was one of the important variants of concern. It gets inside human cells by using its S1 subunit’s receptor-binding domain (SARS-CoV-2-RBD) to bind to Angiotensin-converting enzyme 2 receptor’s peptidase domain (ACE2-PD). Using peptides to inhibit binding interactions (BIs) between ACE2-PD and SARS-CoV-2-RBD is one of promising COVID-19 therapies. Employing computational protein design (CPD) as well as molecular dynamics (MD), this study used ACE2-PD’s α1 helix to generate novel 25-mer peptide binders (SPB25) of Omicron RBD that have predicted binding affinities (ΔG_bind (MM‑GBSA)_) better than ACE2 by increasing favorable BIs between SPB25 and the conserved residues of RBD. Results from MD and the MM-GBSA method identified two best designed peptides (SPB25_T7L/K11A_ and SPB25_T7L/K11L_ with ΔG_bind (MM‑GBSA)_ of −92.4 ± 0.4 and −95.7 ± 0.5 kcal/mol, respectively) that have better ΔG_bind (MM‑GBSA)_ to Omicron RBD than ACE2 (−87.9 ± 0.5 kcal/mol) and SPB25 (−71.6 ± 0.5 kcal/mol). Additionally, they were predicted to have slightly higher stabilities, based on their percent helicities in water, than SBP1 (the experimentally proven inhibitor of SARS-CoV-2-RBD). Our two best designed SPB25s are promising candidates as omicron variant inhibitors.

## Introduction

The coronavirus disease 2019 (COVID-19) pandemic was caused by severe acute respiratory syndrome coronavirus 2 (SARS-CoV-2) and brought about substantial worldwide cases and deaths [1, 2]. This virus has evolved its genome over time, and several variants of concern such as Alpha (B.1.1.7), Beta (B.1.351), Gamma (P.1), Delta (B.1.617.2), and Omicron (B.1.1.529) variants have emerged [3]. Some mutations may modify viral properties, transmissibility, and therapeutic solutions including the performance of vaccines [4]. Structurally, this virus has four principal components such as the spike (S) proteins, envelope (E), nucleocapsid (N) and membrane (M) [5, 6]. The receptor binding (S1) as well as membrane fusion (S2) subunits are in the spike protein. To attach to human cells, this virus uses its S1 subunit’s receptor binding domain (RBD) to bind to angiotensin-converting enzyme 2 (ACE2) receptor’s peptidase domain (PD) in human, while it uses its S2 subunit for the membrane fusion between its membrane and a host membrane [7, 8]. The previous study found that RBDs of Omicron and SARS-CoV-2 bound to monomeric human ACE2 receptor with the dissociation constant (*K*_*D*_) of 38.9 ± 10.5 nM and 75.5 ± 2.1 nM, respectively [9].

To inhibit the virus from entering human cells, blocking binding interactions (BIs) between ACE2-PD and RBD using peptide inhibitors, neutralizing antibodies and small-molecule drugs have been extensively investigated [10–16]. Due to their high structural compatibility with the surface of a protein target, and their abilities to disrupt protein–protein interaction, peptides can be utilized as inhibitors that disrupt ACE2-PD and RBD binding [17, 18].

Computational approaches including molecular dynamics (MD) and computational protein design (CPD) have been utilized for designing peptide binders of RBD of the wuhan variant and other variants [19–22]. To inhibit ACE2 and RBD binding, CPD and MD were employed in our previous studies to design novel 25-mer peptide binders (SPB25), which were derived from ACE2-PD’s α1 helix, that have better predicted SARS-CoV-2-RBD binding affinities (ΔG_bind (MM‑GBSA)_) than ACE2 and 23-mer peptide binder (SBP1) [23, 24], which is the experimentally proven inhibitor of SARS-CoV-2-RBD [25]. Nonetheless, the knowledge on BIs between SPB25 and Omicron RBD as well as on whether our strategy, increasing favorable BIs between SPB25 and the conserved residues of RBD, can be used to create novel 25-mer peptides that have predicted ΔG_bind (MM‑GBSA)_ to Omicron RBD better than ACE2 is limited.

Based on ACE2-PD’s α1 helix (residues 21–45), this study used CPD (Rosetta) as well as MD (AMBER) to generate novel SPB25 that have better predicted ΔG_bind (MM‑GBSA)_ to Omicron RBD than ACE2. We utilized the design strategy of increasing favorable BIs between SPB25 and the conserved residues of RBD. The designed SPB25 that have predicted ΔG_bind (MM‑GBSA)_ to Omicron RBD better than ACE2 are promising peptides that may be utilized to inhibit BI between ACE2 and Omicron RBD.

## Methods

### Preparation of structures

The complex structure of ACE2 binding to Omicron RBD was from PDB ID: 7TN0 [26]. SPB25 structure (21 IEEQAKTFLDKFNHEAEDLFYQSSL 45) binding to Omicron RBD was extracted from ACE2-PD’s α1 helix binding with Omicron RBD (PDB ID: 7TN0 [26]). These complexes were protonated at pH 7.4 (physiological pH) employing the H^++^ server [27]. AMBER18’s LEaP module [28] was utilized to create the final complex structure.

### Computational design of novel SPB25s

In this study, the design template for CPD (Rosetta [29]) was the structure of SPB25 in complex with Omicron RBD. This work employed the design strategy of increasing favorable BIs between the conserved residues of RBD (Y421, L455, F456, G485, F486 and Y489) [30] and residues 21–45 of ACE2 so that the designed SPB25 have better ΔG_bind (MM‑GBSA)_ to Omicron RBD than ACE2. Designed positions were selected if favorable BIs could potentially be formed upon mutations between RBD’s conserved residues and the side chains of designed positions. Employing CoupledMoves protocol [31, 32] of RosettaDesign module (Rosetta3.11) and beta_nov16 energy function, structures of selected designed residues/positions were redesigned, repacked and minimized. Standard amino acids except G and P were allowed in each designed position. Additionally, residues within 10 Å of each designed position were repacked and energetically minimized. 400 runs were independently conducted for each design, producing 400 conformations of designed sequences, where some sequences may have various conformations. To calculate ΔG_bind (Rosetta)_ in Rosetta Energy Unit (REU) of each conformation, an interface analyzer [33, 34] module (Rosetta3.11) was used. ΔΔG_bind (Rosetta)_ upon mutation was determined by deducting ΔG_bind (Rosetta)_ of SPB25 from that of the designed conformation/sequence. MD was performed on designed conformations of all designed positions with ΔΔG_bind (Rosetta)_ < 0 REU, and the molecular mechanics–generalized born surface area (MM-GBSA) method [35–37] was utilized to determine if their ΔG_bind (MM‑GBSA)_ values are better than that of SPB25. The single mutants with better predicted ΔG_bind (MM-GBSA)_ than SPB25 were used to generate the double mutants of SPB25, and subsequently simulated to determine if their ΔG_bind (MM‑GBSA)_ values are superior to that of SPB25.

### MD and analyses

Complex structures of Omicron RBD binding to ACE2 and designed peptides were built in isomeric truncated octahedral boxes of TIP3P water utilizing the buffer distance of 13 Å as well as force field parameters of protein.ff14SB [38] and GLYCAM06j-1 [39] in AMBER18 [28]. To remove interactions that are unfavorable, all systems were energetically minimized, using the five-step procedure [23, 24, 40]. Employing different restraints on proteins, each minimization step contains 2,500 steps of steepest descent and 2,500 steps of conjugate gradient. In the first step, hydrogen atoms and water molecules were energetically minimized, while restraining proteins’ heavy atoms using a force constant of 10 kcal/(mol Å^2^). In the second, third and fourth steps of minimizations, force constants of 10, 5 and 1 kcal/(mol Å^2^) were subsequently applied to restrain proteins’ backbones, respectively. Lastly, the whole system was minimized without any restraining force. Subsequently, the GPU (CUDA) version of PMEMD module [41–43] was used for MD with the periodic boundary condition. To constrain all bonds with hydrogen atoms to allow the time step of 0.002 ps, SHAKE [44] was employed. To control each system’s temperature, the Langevin dynamics method with a collision frequency of 1.0 ps^-1^ was used. For heating, the temperature of each system was increased from 0 K to 310 K in the NVT ensemble during 200 ps MD, while the backbones of the proteins were restrained with the force constant of 10 kcal/(mol Å^2^). Each system was additionally equilibrated in the NVT ensemble without any restraining force at 310 K for 300 ps. In the production run, each system was subject to MD in the NPT ensemble at 310 K and 1 atm for 100 ns.

The structural stabilities of all systems were measured based on their Root Mean Square Deviation (RMSD) values with respect to their minimized structures. Further analyses were conducted on 80–100 ns trajectories, which have stable RMSD values. To predict each complex’s binding affinity, the MM-GBSA technique was utilized to compute their total binding free energies [ΔG_bind (MM-GBSA)_]. The MM-GBSA method uses the following equation to compute ΔG_bind (MM-GBSA)_.

$$\Delta G_{\text{bind}}=G_{\text{complex}}–G_{\text{receptor}}–G_{\text{ligand}}$$

$$=\;\Delta E_{\text{MM}}+\;\Delta G_{\text{polar}\left(\text{GB}\right)}\;+\;\Delta G_{\text{non-polar(SA)}}–T\Delta S$$

$$=\Delta E_{\text{vdw}}+\;\Delta E_{\text{ele}}+\;\Delta G_{\text{polar(GB)}}+\;\Delta G_{\text{non-polar(SA)}}–T\Delta S,$$

where Δ*G*_bind_ is the total binding free energy of the system that is defined as the difference between the free energies of the complex (*G*_complex_), the receptor (*G*_receptor_), and the ligand (*G*_ligand_). Δ*G*_bind_ is calculated as the sum of the molecular mechanics free energy (Δ*E*_MM_), the solvation free energy consisting of polar (Δ*G*_polar(GB)_) and nonpolar contributions (Δ*G*_non-polar(SA)_), and the entropy (–*T*Δ*S*) in the gas phase. The Δ*E*_MM_ term comprises Δ*E*_vdw_ (van der Waals) and Δ*E*_ele_ (electrostatic). The polar and non-polar contributions are estimated by the Generalized-Born (GB) implicit solvation model and the molecular solvent accessible surface area (SASA). In this work, the entropic contribution (–*T*Δ*S*) was not included in the calculation of Δ*G*_bind_ because the nmode module of AMBER predicts this term with high computational cost but not high accuracy [45, 46].

Designed SPB25s with ΔG_bind (MM‑GBSA)_ better than ACE2 were selected for additional analyses including BIs and per-residue free energy decomposition (PFED). Hydrogen-bond (H-bond) occupations were determined to identify H-bonds of all systems. The following rules were employed to consider an occurrence of a H-bond: (i) a proton donor-acceptor distance ≤3.5 Å and (ii) a donor-H-acceptor bond angle ≥120°. In this work, three levels of occupations were defined: (i) strong H-bonds with H-bond occupations > 75%, (ii) medium H-bonds with 75% ≥ H-bond occupations > 50%, and (iii) weak H-bond interactions with 50% ≥ H-bond occupations > 25% [23, 24, 40, 47]. To measure the peptide helicity of each system, Define Secondary Structure of Protein (DSSP) was utilized to compute the percent helicity from the summation of the percentage of α-, π- and 3-10-helix structures [48].

## Results

### Computational design of SPB25s of Omicron RBD

**Fig 1:**
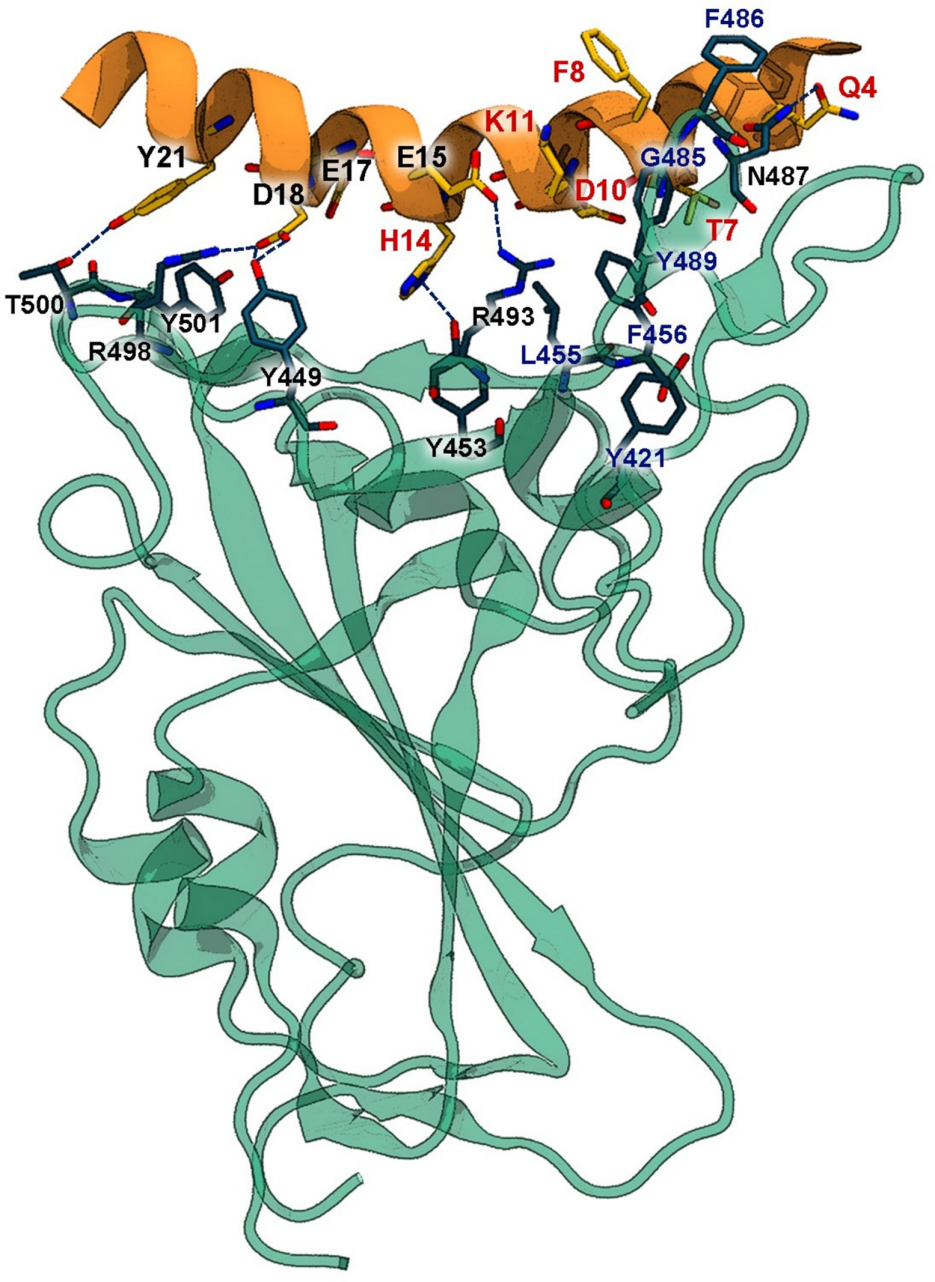
The design template structure of SPB25 binding to Omicron RBD. Omicron RBD and SPB25 are displayed in cyan and orange, respectively. Conserved residues of RBD and the designed positions are labelled in blue and red, respectively.

The template structure of SPB25 binding to Omicron RBD (Fig 1) was extracted from the structure of ACE2-PD’s α1 helix binding to Omicron RBD (PDB ID: 7TN0). Our design strategy is enhancing favorable BIs between the conserved residues of RBD (Y421, L455, F456, G485, F486 and Y489) [30] and SPB25. Designed positions of SPB25 were selected if favorable BIs could potentially form upon mutations between their side chains and the conserved residues of RBD. Q4(24), T7(27), F8(28), D10(30), K11(31) and H14(34) were chosen based on this criterion. Standard amino acids, except G and P due to their low occurring frequencies in an α-helix, were allowed in each designed position. In addition, P could cause the formation of a kink that can destabilize a helix [49]. Using these designed positions and amino acid types as inputs to Rosetta, 52 designed SPB25s that have single mutations (S1 Table) were produced. Sixteen designed SPB25s with better ΔG_bind (Rosetta)_ than SPB25 (ΔΔG_bind (Rosetta)_ < 0 REU) were simulated to determine if their ΔG_bind (MM‑GBSA)_ calculated by the more accurate MM-GBSA method were better than that of SPB25 (ΔΔG_bind (MM‑GBSA)_ < 0 kcal/mol). SPB25_Q4C_, SPB25_T7L_, SPB25_T7W_, SPB25_F8A_, SPB25_D10L_, SPB25_D10M_, SPB25_D10R_, SPB25_K11A_, SPB25_K11L_, SPB25_K11M_, SPB25_K11N_, SPB25_K11Q_, SPB25_K11V_, SPB25_K11W_, SPB25_K11Y_, and SPB25_H14V_ are designed peptides with ΔΔG_bind (MM‑GBSA)_ < 0 kcal/mol.

### MD and calculations of binding free energies

Complex structures of all 16 designed 25-mer peptides, SPB25 and ACE2 binding to Omicron RBD were simulated for 100 ns. Using the MM-GBSA technique, ΔG_bind (MM-GBSA)_ of all complexes were computed to assess whether ΔG_bind (MM‑GBSA)_ of designed SPB25s are better than that of SPB25. To determine the stabilities of each system, Root Mean Square Deviation (RMSD) of all and backbone atoms were computed (S1 Fig). RMSD plots show that it likely took around 80 ns for each system to reach equilibrium. As a result, further analyses were conducted on the 80–100 ns trajectory of each system.

The MM-GBSA technique was utilized to compute ΔG_bind (MM‑GBSA)_ of each system from its 80−100 ns trajectory. Table 1 illustrates that ΔG_bind (MM‑GBSA)_ of ACE2 and SPB25 binding to Omicron RBD are −87.9 ± 0.5, and −71.6 ± 0.5 kcal/mol, respectively. ΔG_bind (MM-GBSA)_ of seven designed SPB25s, out of 16 designed SPB25s with single mutations, such as SPB25_T7L_, SPB25_F8A_, SPB25_K11A_, SPB25_K11L_, SPB25_K11M_, SPB25_K11Q_, and SPB25_K11V_ are better than that of SPB25 with ΔΔG_bind (MM-GBSA)_ of −3.1 ± 0.8, −14.9 ± 0.8, −1.6 ± 0.7, −9.2 ± 0.7, −7.8 ± 0.8, −5.8 ± 0.8, and −9.6 ± 0.9 kcal/mol, respectively. The total of 11 and 5 designed SPB25s containing double and triple mutations were also built based on the seven designed SPB25s with single mutations, employing Rosetta. They were subject to MD, and their values of ΔG_bind (MM-GBSA)_ were predicted by the MM-GBSA technique. In terms of designed SPB25s that contain double mutations, ΔG_bind (MM-GBSA)_ of SPB25_T7L/F8A_, SPB25_T7L/K11A_, SPB25_T7L/K11L_, SPB25_T7L/K11Q_, SPB25_T7L/K11V_, SPB25_F8A/K11L_, SPB25_F8A/K11M_, SPB25_F8A/K11Q_, and SPB25_F8A/K11V_ are better than that of SPB25 with the ΔΔG_bind (MM-GBSA)_ of −13.0 ± 0.9, −20.8 ± 0.6, −24.1 ± 0.7, −6.5 ± 0.7, −7.4 ± 0.8, −2.1 ± 0.7, −3.1 ± 0.7, −4.5 ± 0.8, and −6.2 ± 0.9 kcal/mol, respectively. Moreover, the predicted binding affinities of SPB25_T7L/K11A_ (ΔG_bind (MM-GBSA)_ = −92.4 ± 0.4 kcal/mol) and SPB25_T7L/K11L_ (ΔG_bind (MM-GBSA)_ = −95.7 ± 0.5 kcal/mol) are better than that of ACE2 (ΔG_bind (MM-GBSA)_ = −87.9 ± 0.5 kcal/mol). For designed SPB25s with triple mutations, only ΔG_bind (MM-GBSA)_ of SPB25_T7L/F8A/K11Q_ is better than that of SPB25 with ΔΔG_bind (MM-GBSA)_ of −5.2 ± 0.8 kcal/mol.

**Table 1:** Predicted binding free energies, by Rosetta and the MM-GBSA technique, to Omicron RBD of ACE2, SPB25 and designed SPB25 that were chosen for simulations.

System	ΔΔG_bind (Rosetta)_^a^ (REU)	ΔG_bind (MM-GBSA)_ (kcal/mol)	ΔΔG_bind (MM-GBSA)_^b^ (kcal/mol)
ACE2	-	−87.9 ± 0.5	−16.3 ± 0.7
SPB25	-	−71.6 ± 0.5	0.0 ± 0.7
SPB25_Q4C_	−0.2	−65.7 ± 0.7	5.9 ± 0.9
SPB25_T7L_	−1.9	−74.7 ± 0.6	−3.1 ± 0.8
SPB25_T7W_	−0.9	−61.6 ± 0.7	10.0 ± 0.9
SPB25_F8A_	−0.2	−86.5 ± 0.6	−14.9 ± 0.8
SPB25_D10L_	−0.8	−70.1 ± 0.8	1.5 ± 0.9
SPB25_D10M_	−1.7	−70.0 ± 0.7	1.6 ± 0.9
SPB25_D10R_	−2.4	−69.8 ± 0.5	1.8 ± 0.7
SPB25_K11A_	−0.4	−73.2 ± 0.5	−1.6 ± 0.7
SPB25_K11L_	−0.6	−80.8 ± 0.5	−9.2 ± 0.7
SPB25_K11M_	−0.1	−79.4 ± 0.6	−7.8 ± 0.8
SPB25_K11N_	−0.8	−66.2 ± 0.4	5.4 ± 0.6
SPB25_K11Q_	−0.1	−77.4 ± 0.6	−5.8 ± 0.8
SPB25_K11V_	−1.9	−81.2 ± 0.7	−9.6 ± 0.9
SPB25_K11W_	−0.8	−61.3 ± 0.4	10.3 ± 0.6
SPB25_K11Y_	−1.1	−61.9 ± 0.5	9.7 ± 0.7
SPB25_H14V_	−0.7	−65.4 ± 0.5	6.2 ± 0.7
SPB25_T7L/F8A_	−0.8	−84.6 ± 0.7	−13.0 ± 0.9
SPB25_T7L/K11A_	0.9	−92.4 ± 0.4	−20.8 ± 0.6
SPB25_T7L/K11L_	−2.1	−95.7 ± 0.5	−24.1 ± 0.7
SPB25_T7L/K11M_	−0.1	−57.0 ± 0.7	14.6 ± 0.9
SPB25_T7L/K11Q_	−1.1	−78.1 ± 0.5	−6.5 ± 0.7
SPB25_T7L/K11V_	−1.9	−79.0 ± 0.6	−7.4 ± 0.8
SPB25_F8A/K11A_	−0.1	−71.0 ± 0.4	0.6 ± 0.6
SPB25_F8A/K11L_	−2.8	−73.7 ± 0.5	−2.1 ± 0.7
SPB25_F8A/K11M_	−0.8	−74.7 ± 0.5	−3.1 ± 0.7
SPB25_F8A/K11Q_	−2.0	−76.1 ± 0.6	−4.5 ± 0.8
SPB25_F8A/K11V_	−2.1	−77.8 ± 0.7	−6.2 ± 0.9
SPB25_T7L/F8A/K11A_	0.4	−51.6 ± 0.8	20.0 ± 0.9
SPB25_T7L/F8A/K11L_	−3.1	−59.6 ± 0.4	12.0 ± 0.6
SPB25_T7L/F8A/K11M_	−1.6	−59.1 ± 0.6	12.5 ± 0.8
SPB25_T7L/F8A/K11Q_	−1.5	−76.8 ± 0.6	−5.2 ± 0.8
SPB25_T7L/F8A/K11V_	−1.9	−67.0 ± 0.5	4.6 ± 0.7

^a^ The difference between ΔG_bind (Rosetta)_ of a system and that of SPB25.

^b^ The difference between ΔG_bind (MM-GBSA)_ of a system and that of SPB25.

### The binding free energy components of designed SPB25s with better ΔG_bind (MM‑GBSA)_ than ACE2

**Fig 2:**
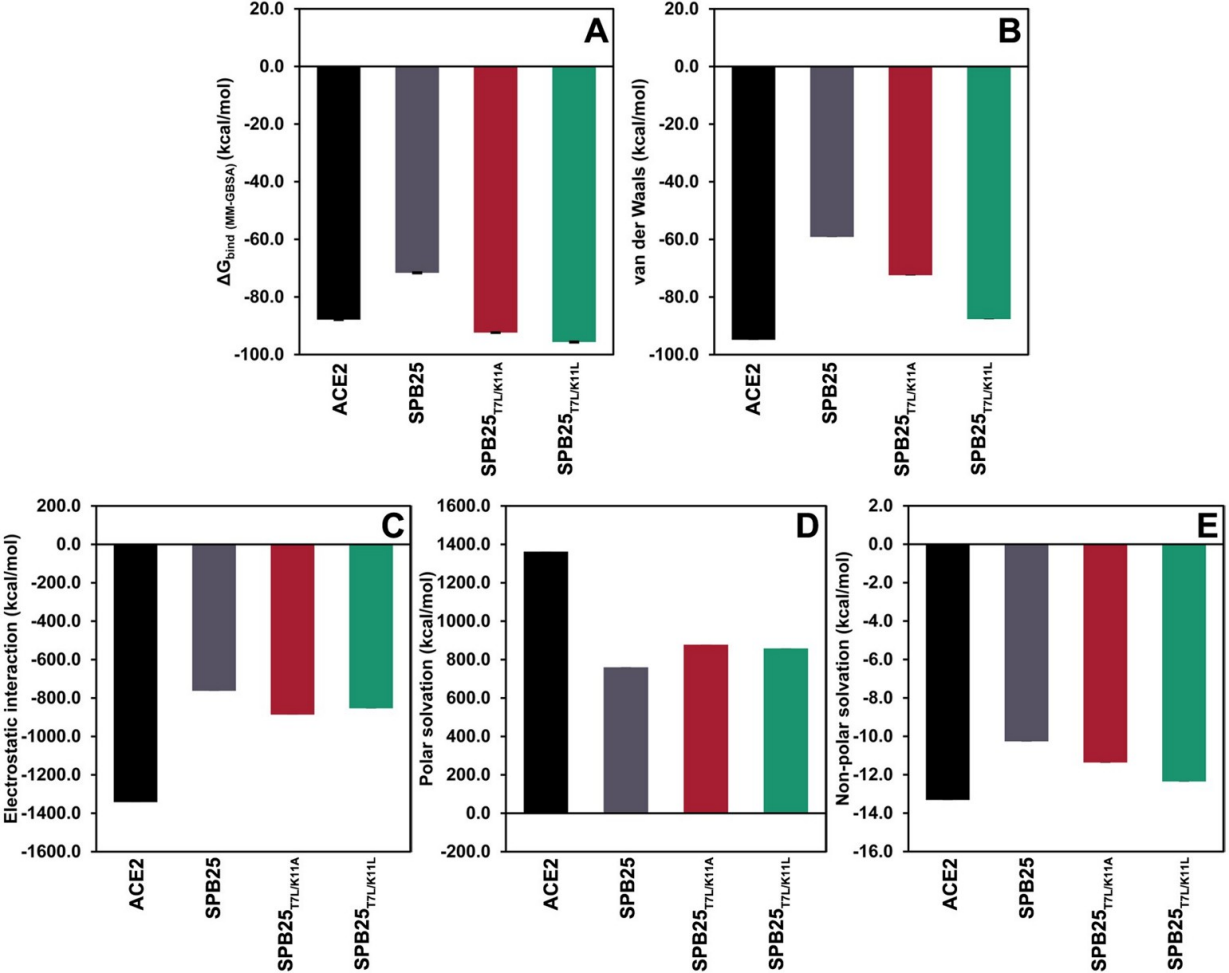
The binding free energy components of ACE2, SPB25 and designed SPB25s in complex with Omicron RBD. A) ΔG_bind (MM-GBSA)_, B) van der Waals energy, C) electrostatic interaction D) polar solvation and E) non-polar solvation.

The binding free energy components of ACE2, SPB25 and two designed SPB25s with better predicted ΔG_bind (MM‑GBSA)_ to Omicron RBD than ACE2 are shown in Fig 2. The main contributor to the favorable ΔG_bind (MM-GBSA)_ to Omicron RBD of ACE2, SPB25, SPB25_T7L/K11A_, and SPB25_T7L/K11L_ is the electrostatic interaction term. Other terms that also favorably contribute to ΔG_bind (MM‑GBSA)_ are the non-polar solvation and van der Waals energy terms. The term that unfavorably contributes to ΔG_bind (MM‑GBSA)_ is the polar solvation term.

SPB25_T7L/K11A_ and SPB25_T7L/K11L_ are the designed 25-mer peptides that have the best ΔG_bind (MM‑GBSA)_ to Omicron RBD (ΔG_bind (MM-GBSA)_ values of −92.4 ± 0.4 and −95.7 ± 0.5 kcal/mol, respectively). Their ΔG_bind (MM‑GBSA)_ are better than that of SPB25 by 20.8 ± 0.6 and 24.1 ± 0.7 kcal/mol, respectively, and better than that of ACE2 by 4.5 ± 0.6 and 7.8 ± 0.7 kcal/mol, respectively. As compared to those of SPB25, the major contributions to the favorable BI of SPB25_T7L/K11A_ and SPB25_T7L/K11L_ to Omicron RBD are the increases in their favorable electrostatic interaction, van der Waals energy, and non-polar solvation terms. Nevertheless, their unfavorable polar solvation terms are higher than that of SPB25.

### Elucidation of important binding residues (IBRs) of designed SPB25s with ΔG_bind (MM-GBSA)_ better than ACE2

**Fig 3:**
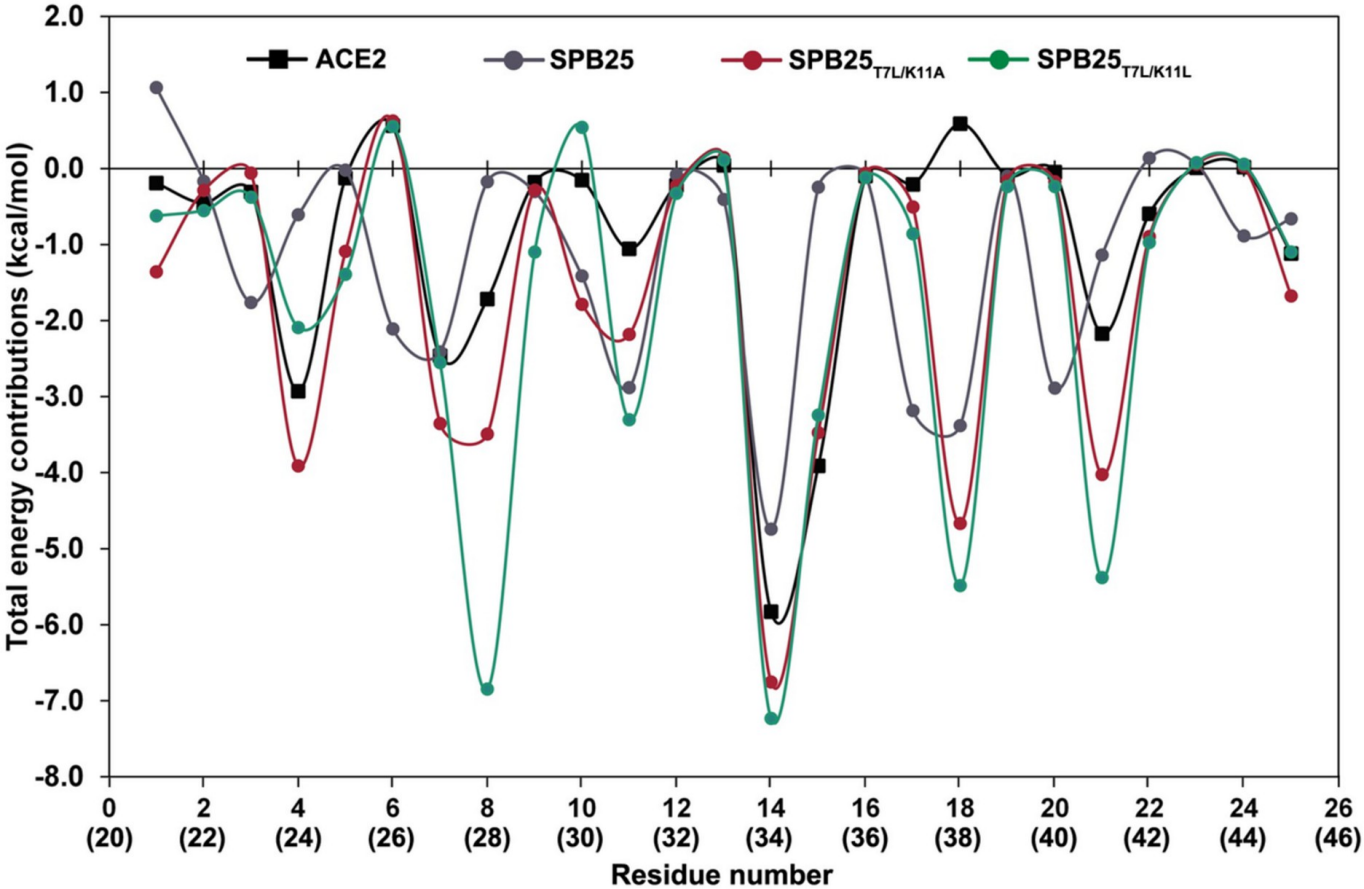
PFED of the best designed 25-mer peptides, SPB25, and ACE2 in binding to Omicron RBD. ACE2’s residue numbers are shown in parenthesis.

To elucidate IBRs to Omicron RBD, PFED of two designed 25-mer peptides with ΔG_bind (MM-GBSA)_ better than ACE2 were computed (Fig 3). Residues that have the total energy contribution (TEC) better than −1.0 kcal/mol were identified as IBRs. In terms of residues 21–45 of the α1 helix of ACE2, Q24, T27, F28, K31, H34, E35, Y41, and L45 were identified as IBRs to Omicron RBD. The predicted IBRs of SPB25 are E3(23), K6(26), T7(27), D10(30), K11(31), H14(34), E17(37), D18(38), F20(40), and Y21(41). The total IBRs of SPB25_T7L/K11A_ (12) and SPB25_T7L/K11L_ (11) are more than those of residues 21–45 of the α1 helix of ACE2 (8) and SPB25 (10). Eight residues of these two designed 25-mer peptides have high predicted ΔG_bind (MM‑GBSA)_ (better than -2 kcal/mol) including Q4, L7, F8, A11/L11, H14 (the highest binding affinity residue), E15, D18 and Y21.

In terms of TECs of SPB25_T7L/K11A_, the T7L/K11A mutations were predicted to favorably enhance TECs of residue 7 from −2.5 and −2.4 kcal/mol in ACE2 and SPB25, respectively to −3.4 in SPB25_T7L/K11A_. TEC of residue 11 was favorably enhanced from −1.1 kcal/mol in ACE2 to −2.2 kcal/mol in SPB25_T7L/K11A_, while it was unfavorably reduced from −2.9 kcal/mol in SPB25 to −2.2 kcal/mol in SPB25_T7L/K11A_. Nonetheless, TECs of other residues including I1, Q4, A5, F8, D10, H14, D18, Y21 and L25 were favorably enhanced from −0.2, −2.9, −0.1, −1.7, −0.2, −5.8, 0.6, −2.2 and −1.1 kcal/mol in ACE2 and 1.1, −0.6, 0.0, −0.2, −1.4, −4.7, −3.4, −1.1 and −0.7 kcal/mol in SPB25 to −1.4, −3.9, −1.1, −3.5, −1.8, −6.8, −4.7, −4.0 and −1.7 kcal/mol in SPB25_T7L/K11A_, respectively. Additionally, TEC of E15 was also favorably enhanced from −0.2 kcal/mol in SPB25 to −3.5 kcal/mol in SPB25_T7L/K11A_.

In terms of TECs of SPB25_T7L/K11L_, the T7L/K11L mutations were predicted to favorably enhance TECs of residues 7 and 11 from −2.5 and −1.1 kcal/mol in ACE2 and −2.4 and −2.9 kcal/mol in SPB25 to −2.6 and −3.3 kcal/mol in SPB25_T7L/K11L_, respectively, Additionally, TECs of A5, F8, L9, H14, D18 and Y21 were also favorably enhanced from −0.1, −1.7, −0.2, −5.8, 0.6 and −2.2 kcal/mol in ACE2 and 0.0, −0.2, −0.3, −4.7, −3.4 and −1.1 kcal/mol in SPB25 to −1.4, −6.8, −1.1, −7.2, −5.5 and −5.4 kcal/mol in SPB25_T7L/K11L_, respectively. Moreover, TECs of Q4, E15 and L25 were favorably enhanced from −0.6, −0.2 and −0.7 kcal/mol in SPB25 to −2.1, −3.2 and −1.1 kcal/mol in SPB25_T7L/K11L_.

### H-bond and π interactions of designed SPB25s that have better ΔG_bind (MM‑GBSA)_ than ACE2

To elucidate important H-bonds, cation-π, anion-π, π-π, σ-π and alkyl-π interactions for the binding of ACE2, SPB25 and the best 25-mer designed peptides to Omicron RBD, H-bond occupations and π interactions were investigated as shown in Tables 2 and S2. H34, Y83 and K353 of ACE2 had strong H-bonds with the backbones of R493, N487 and G502 of Omicron RBD, respectively. S19, Q24, E35, D38 and D355 of ACE2 had medium H-bonds with A475, N487, R493, Y449 and T500 of Omicron RBD. Additionally, there were weak H-bonds between S19, H34, E35 and Y41 of ACE2 and A475, S494, R493 and T500 of Omicron RBD, respectively. For the π interactions between Omicron RBD and ACE2, there was a cation-π interaction between K31 of ACE2 and Y489 of Omicron RBD. F28, H34, Y41 and Y83 of ACE2 had π-π interactions with F486, Y453, Y501 and F486 of Omicron RBD. There was also a σ-π interaction between H34 of ACE2 and R493 of Omicron RBD. In addition, K31, H34, L79, M82 and K353 of ACE2 had alkyl-π interactions with Y489, R493, F486, F486, Y501 and H505 of Omicron RBD, respectively.

**Table 2:** No. of H-bond and π interactions of ACE2, SPB25 and designed SPB25s involved in binding to Omicron RBD.

System	No. of H-bonds			Residue that forms a H-bond with Omicron RBD	π interaction				
	Strong	Medium	Weak		Cation-π	Anion-π	π-π	σ-π	Alkyl-π
ACE2	4	7	5	S19, Q24, H34, E35, D38, Y41, Y83, K353, D355	K31@NZ–Y489	-	F28 –F486 H34 –Y453 Y41 –Y501 Y83 –F486	H34 –R493@HB3	K31 –Y489 H34 –R493 L79 –F486 M82 –F486 K353 –Y501 K353 –H505
SPB25	6	3	2	T7, D10, H14, E17, D18	H14 –R493@NH1 Y21 –R493@NH2	-	H14 –F456 H14 –Y489 F20 –Y501 F20 –H505	-	K11 –Y489 H14 –L455
SPB25_T7L/K11A_	7	4	9	Q4, D10, H14, E15, D18, Y21	H14 –R403@NH1 H14 –R493@NH2	-	F8 –Y489 H14 –Y501 H14 –H505 Y21 –Y501 Y21 –H505	Y21 –Y501@HA	A5 –Y489 H14 –R493
SPB25 _T7L/K11L_	6	1	6	Q4, H14, E15, D18, Y21	-	E17@OE1 –H505 E17@OE2 –H505	F8 –F456 F8 –Y473 F8 –Y489 H14 –Y453 H14 –Y495 H14 –H505 Y21 –Y501 Y21 –H505	Y21 –Y501@HA	A5 –Y473 A5 –Y489 L7 –F456 F8 –L455 F8 –A475 L9 –Y489 H14 –R493

For H-bond and π interactions between SPB25 and Omicron RBD, D10, H14, E17 and D18 of SPB25 had six strong H-bonds with N417, R493, R403 and R493 of Omicron RBD. There were also medium H-bonds between T7, D10 and E17 of SPB25 and Y473, Y421 and R403 of Omicron RBD, respectively. Additionally, there were two weak H-bonds between E17 and E18 of SPB25 and R403 and R493 of Omicron RBD. In terms of π-interactions, H14 and Y21 of SPB25 had two cation-π interactions with R493 of Omicron RBD. There were four π-π interactions between H14 and F20 of SPB25 and F456, Y489, Y501 and H505 of Omicron RBD. Moreover, K11 and H14 of SPB25 had alkyl-π interactions with Y489 and L455 of Omicron RBD, respectively.

**Fig 4:**
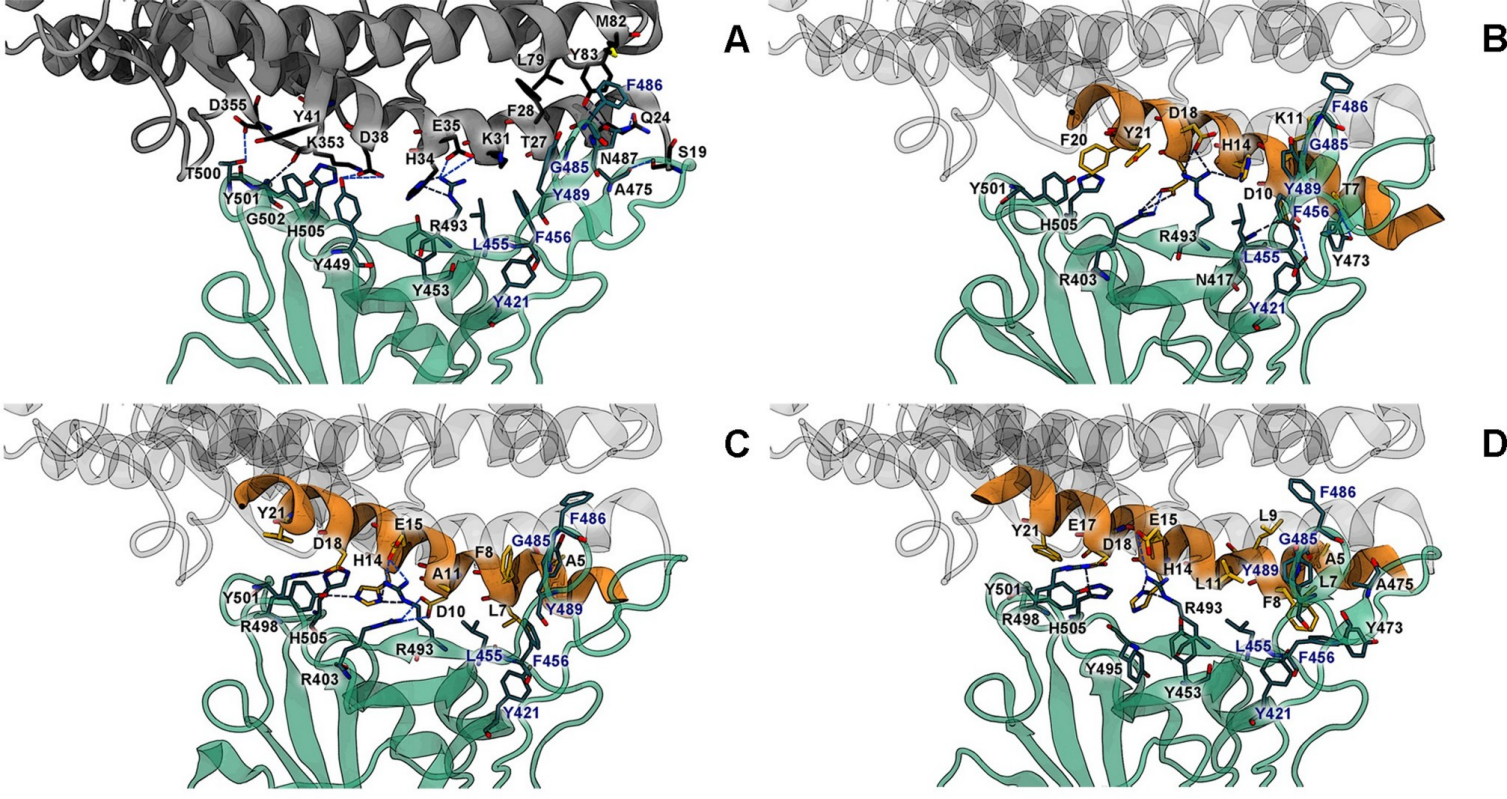
Key BIs between Omicron RBD (cyan) and A) ACE2, B) SPB25, C) SPB25_T7L/K11A_, or D) SPB25_T7L/K11L_. The structures closest to the average structures from the 80–100 ns MD trajectories of SPB25 and designed SPB25s (orange) were superimposed with that of ACE2 (grey). Key salt bridges and H-bonds (H-bond occupations > 25%) are represented as blue dashed lines.

Fig 4 illustrates key predicted BIs between the two best designed 25-mer peptides and Omicron RBD. The overall binding poses of these two designed SPB25s and ACE2 binding to omicron RBD are relatively similar. In terms of predicted interactions to Omicron RBD, the total no. of H-bonds of SPB25_T7L/K11A_ is higher than those of ACE2 and SPB25. Additionally, no. of strong H-bonds of SPB25_T7L/K11A_ is higher than those of ACE2 and SPB25, supporting the result that its ΔG_bind (MM‑GBSA)_ is better than ACE2 and SPB25. D18 of SPB25_T7L/K11A_ had four strong H-bonds with R498 and Y501 of Omicron RBD. Additionally, H14 of SPB25_T7L/K11A_ had three strong H-bonds with R493 and Y501 of Omicron RBD. D10 and E15 of SPB25_T7L/K11A_ had four medium H-bonds with R403 and R493 of Omicron RBD. Furthermore, other residues such as Q4 and Y21 had H-bonds with Omicron RBD. In terms of π interactions between SPB25_T7L/K11A_ and Omicron RBD, no. of π interactions of SPB25_T7L/K11A_ is more than that of SPB25. H14 of SPB25_T7L/K11A_ had two cation-π interactions with R403 and R493 of Omicron RBD. In addition, there were five π-π interactions (F8 –Y489, H14 –Y501, H14 –H505, Y21 –Y501 and Y21 –H505), one σ-π interaction (Y21 –Y501@HA), and two alkyl-π interactions (A5 –Y489 and H14 –R493) between SPB25_T7L/K11A_ and Omicron RBD.

Regarding the predicted BIs of SPB25_T7L/K11L_, the total no. of H-bonds of SPB25_T7L/K11L_ is higher than that of SPB25 and lower than that of ACE2. However, no. of π interactions of SPB25_T7L/K11L_ is higher than those of ACE2 and SPB25. H14 and D18 of SPB25_T7L/K11L_ had six strong H-bonds with R493, R498 and Y501 of Omicron RBD. Moreover, there was one medium H-bond between E15 of SPB25_T7L/K11L_ and R493 of Omicron RBD. Additionally, Q4 and Y21 had H-bonds with Omicron RBD. The mutated residue L7 of SPB25_T7L/K11L_ also had alkyl-π interaction with F456 of Omicron RBD, while T27 of ACE2 and T7 of SPB25 had no π interaction with Omicron RBD. Furthermore, other residues had two anion-π interactions (E17@OE1 –H505 and E17@OE2 –H505), eight π-π interactions (F8 –F456, F8 –Y473, F8 –Y489, H14 –Y453, H14 –Y495, H14 –H505, Y21 –Y501 and Y21 –H505), one σ-π interaction (Y21 –Y501@HA), and seven alkyl-π interactions (A5 –Y473, A5 –Y489, L7 –F456, F8 –L455, F8 –A475, L9 –Y489, H14 –R493) between SPB25_T7L/K11L_ and Omicron RBD.

### Peptide helicities of designed SPB25s with better ΔG_bind (MM‑GBSA)_ than ACE2

**Fig 5:**
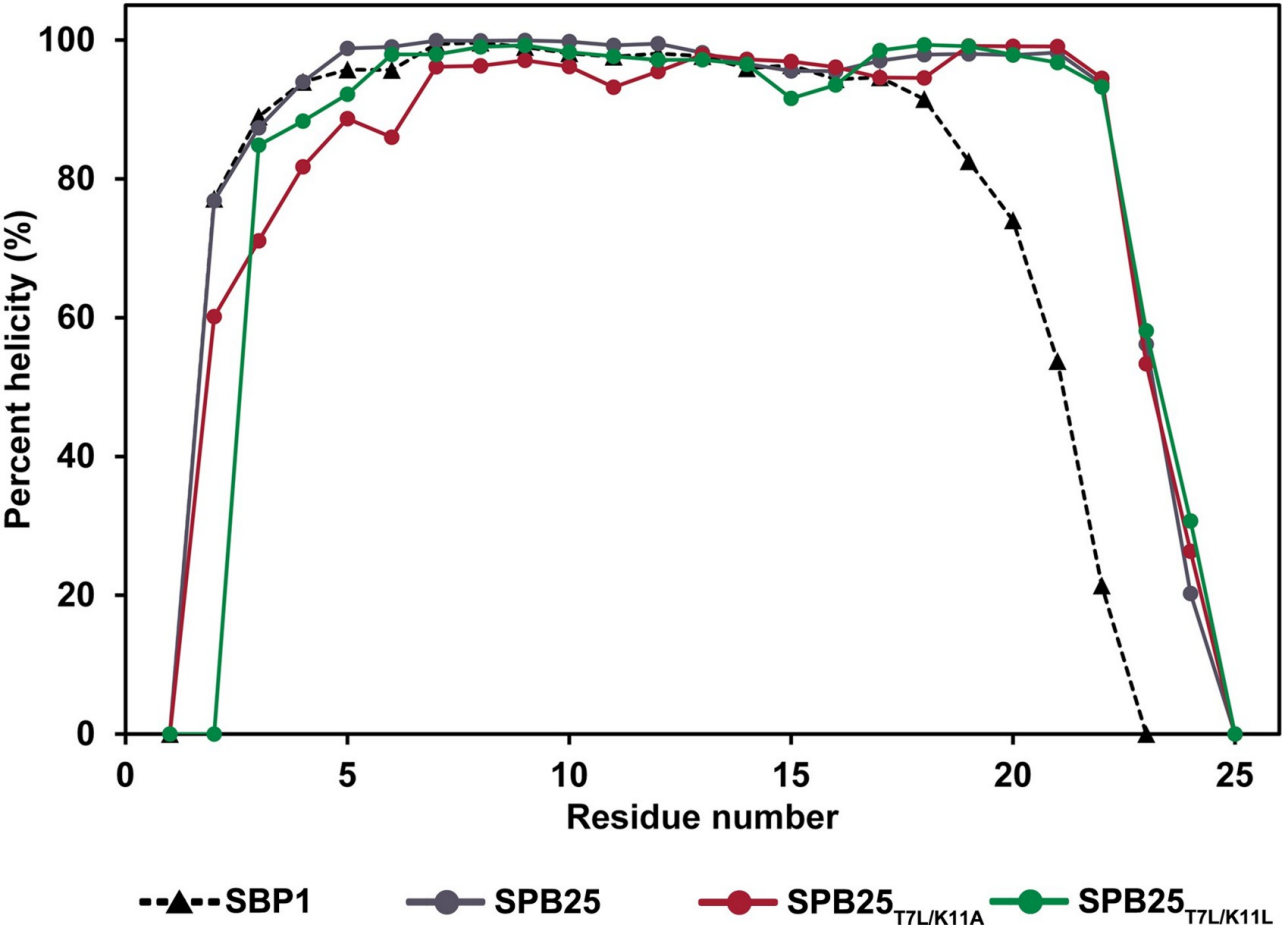
The percent helicities in water of SBP1 [23], SPB25 and designed SPB25s with better ΔG_bind (MM-GBSA)_ than ACE2.

Figs 5 and S2 show the percent helicities and structural stabilities (RMSD plots) in water of designed 25-mer peptides with better predicted bind affinities to Omicron RBD than ACE2, respectively. The percent helicities of the N- and C-termini of the two best designed peptides are lower than those of middle residues due to their high flexibilities. Overall trends of percent helicities of SPB25_T7L/K11A_ and SPB25_T7L/K11L_ are slightly higher than those of SBP1.

## Discussion

The omicron variant (B.1.1.529) was the important variant of concern that was responsible for the COVID-19 pandemic. Similar to other variants, Omicron RBD firstly attaches to ACE2-PD to enter human cells. Disrupting BIs between ACE2-PD and Omicron RBD to prevent coronavirus from infecting and destroying human cells is a promising COVID-19 therapy. Since peptides have more similar interactions to native protein-protein interactions and functional groups than small molecules, which can be ineffective in disrupting large protein-binding interfaces[17, 18], peptides can be employed as inhibitors of SARS-CoV-2 to inhibit protein-protein interactions at their binding interfaces.

To design novel 25-mer peptides with high potential to bind to Omicron RBD better than ACE2, we employed CPD, using the residues 21–45 of ACE2-PD’s α1 helix as a template, and MD. The design strategy of this study was increasing favorable BIs between SPB25 and the conserved residues of RBD (Y421, L455, F456, G485, F486 and Y489). Q4(24), T7(27), F8(28), D10(30), K11(31) and H14(34) were chosen as designed positions because their side chains are in the orientations that can possibly form favorable BIs with Omicron RBD upon mutations. Standard amino acids, except G and P, were allowed for all designed positions. After CPD by Rosetta, 52 designed SPB25s that have single mutations were generated. Sixteen designed SPB25s with superior ΔG_bind (Rosetta)_ to SPB25 (ΔΔG_bind (Rosetta)_ < 0 REU) were simulated, and their ΔG_bind (MM‑GBSA)_ were computed by the MM-GBSA technique and compared to those of SPB25 and ACE2. The predicted binding affinity of ACE2 to Omicron RBD (ΔG_bind (MM-GBSA)_ = −87.9 ± 0.5 kcal/mol) is better than that of SARS-CoV-2-RBD (ΔG_bind (MM-GBSA)_ = −71.2 ± 0.4 kcal/mol) [23], supporting the experimental findings that ACE2 bound to Omicron RBD (K_D_ = 38.9 ± 10.5 nM) with higher affinity than the wild type (K_D_ = 75.5 ± 2.1 nM) [9]. Additionally, the predicted binding affinity of SPB25 to Omicron RBD (−71.6 ± 0.5 kcal/mol) is better than that of SARS-CoV-2-RBD (−60.3±0.4 kcal/mol) [23], suggesting that it should be able to experimentally bind to Omicron RBD better than that of the wild type. However, the predicted binding affinity to Omicron RBD of SPB25 is worse than that of ACE2 probably because no. of IBRs of ACE2 to Omicron RBD is significantly more than SPB25, which also includes those in the α2-helix and the linker of the β3- and β4-sheets.

The predicted binding affinities to Omicron RBD of seven designed 25-mer peptides such as SPB25_T7L_, SPB25_F8A_, SPB25_K11A_, SPB25_K11L_, SPB25_K11M_, SPB25_K11Q_, and SPB25_K11V_ are better than that of SPB25 with ΔΔG_bind (MM-GBSA)_ of −3.1 ± 0.8, −14.9 ± 0.8, −1.6 ± 0.7, −9.2 ± 0.7, −7.8 ± 0.8, −5.8 ± 0.8, and −9.6 ± 0.9 kcal/mol, respectively. They were subsequently used to create 11 and 5 designed peptides that have double and triple mutations, respectively, using Rosetta. Subsequently, all designed peptides were subjected to MD, and their ΔG_bind (MM-GBSA)_ were calculated. For the designed 25-mer peptides with double mutations, the predicted binding affinities to Omicron RBD of SPB25_T7L/F8A_, SPB25_T7L/K11A_, SPB25_T7L/K11L_, SPB25_T7L/K11Q_, SPB25_T7L/K11V_, SPB25_F8A/K11L_, SPB25_F8A/K11M_, SPB25_F8A/K11Q_, and SPB25_F8A/K11V_ are better than that of SPB25 with ΔΔG_bind (MM-GBSA)_ of −13.0 ± 0.9, −20.8 ± 0.6, −24.1 ± 0.7, −6.5 ± 0.7, −7.4 ± 0.8, −2.1 ± 0.7, −3.1 ± 0.7, −4.5 ± 0.8, and −6.2 ± 0.9 kcal/mol, respectively. In terms of designed SPB25s containing triple mutations, the predicted binding affinity to Omicron RBD of SPB25_T7L/F8A/K11Q_ is better than that of SPB25 with ΔΔG_bind (MM-GBSA)_ of −5.2 ± 0.8 kcal/mol. Most importantly, the predicted binding affinities to Omicron RBD of two designed peptides (SPB25_T7L/K11A_ and SPB25_T7L/K11L_) are better than that of ACE2 by 4.5 ± 0.6 and 7.8 ± 0.7 kcal/mol, respectively, suggesting that they should be able to experimentally bind to Omicron RBD better than ACE2. Furthermore, the binding positions and orientations to Omicron RBD of all designed SPB25s and those of residues 21–45 of ACE2-PD’s α1 helix are relatively similar, suggesting that they could potentially inhibit Omicron RBD and ACE2-PD binding.

Our best designed SPB25 is SPB25_T7L/K11L_ since its ΔG_bind (MM-GBSA)_ is better than those of ACE2, SPB25 and SPB25_T7L/K11A_. This result is supported by the fact that no. of π interactions (including A5, L7, F8, L9, H14, E17 and Y21) is higher than those of ACE2, SPB25 and SPB25_T7L/K11A_. Its total no. of H-bonds (including Q4, H14, E15, D18 and Y21) is more than that of SPB25 and lower than that of ACE2. Nonetheless, its no. of strong H-bonds is higher than that of ACE2. The results from PFED suggest Q4, A5, L7, F8, L9, A11, H14, E15, D18, Y21 and L25 as IBRs. Moreover, the T7L/K11L mutation was predicted to favorably enhance TEC of residue 7 and 11 and other residues such as A5, F8, L9, H14, D18 and Y21 as compared to those of ACE2 and SPB25.

ΔG_bind (MM-GBSA)_ of SPB25_T7L/K11A_ is better than those of SPB25 and ACE2, and this finding is supported by the fact that the total no. of H-bonds (including Q4, D10, H14, E15, D18 and Y21) is higher than those of ACE2 and SPB25. Its no. of strong H-bonds (including H14 and D18) is also higher than those of ACE2 and SPB25. Furthermore, its total no. of π interactions (including A5, F8, H14 and Y21) is more than that of SPB25. PFED analysis suggests I1, Q4, A5, L7, F8, D10, A11, H14, E15, D18, Y21 and L25 as IBRs. Additionally, the T7L/K11A mutation was predicted to bring about substantial enhancement in TEC of residue 7 and other residues such as I1, Q4, A5, F8, D10, H14, D18, Y21 and L25 as compared to those of ACE2 and SPB25.

The trends of percent helicities in water of SPB25_T7L/K11A_ and SPB25_T7L/K11L_ are relatively similar to SPB25 [23] and slightly higher than that of SBP1 [23]. Our findings suggest that their stabilities in water may be relatively similar to SPB25 and slightly higher than that of SBP1, and the stabilities of these designed SPB25 should be sufficient for their use as peptide binders.

Using the combination of CPD and MD, we designed promising SPB25s with better ΔG_bind (MM‑GBSA)_ to Omicron RBD than human ACE2 receptor and SPB25 by increasing favorable BIs between peptides and the conserved residues of RBD. The results from MD and the MM-GBSA calculation show that the values of ΔG_bind (MM-GBSA)_ of two best designed peptides (SPB25_T7L/K11A_ and SPB25_T7L/K11L_) are better than those of ACE2 and SPB25. Moreover, their predicted helicities in water are slightly higher than that of SBP1, suggesting that their stabilities are higher than that of SBP1. SPB25_T7L/K11A_ and SPB25_T7L/K11L_ are promising peptide candidates that could possibly be utilized to disrupt BIs between Omicron RBD and ACE2.

## Supporting information

S1 Fig RMSD plots of ACE2, SPB25 and designed peptides in complex with RBD of the omicron variant. The RMSD values of all atoms and backbone atoms are shown in black and grey respectively.
(TIF)

S2 Fig RMSD plots of SPB25 and designed peptides in water. The RMSD values of all atoms and backbone atoms are shown in black and grey respectively.
(TIF)

S1 Table The binding free energies of ACE2, SPB25 and all designed peptides to RBD of the omicron variant as calculated by Rosetta and MM-GBSA method. (PDF)

S2 Table Hydrogen bond occupations of ACE2, SPB25 and designed peptides binding to RBD of the omicron variant. (PDF)
