# Supplementary figures and images for: Designing peptides predicted to bind to the omicron variant better than ACE2 via computational protein design and molecular dynamics
Source: PLoS One. 2023 Oct 10;18(10):e0292589. doi: 10.1371/journal.pone.0292589 (PMC10564162; doi:10.1371/journal.pone.0292589)

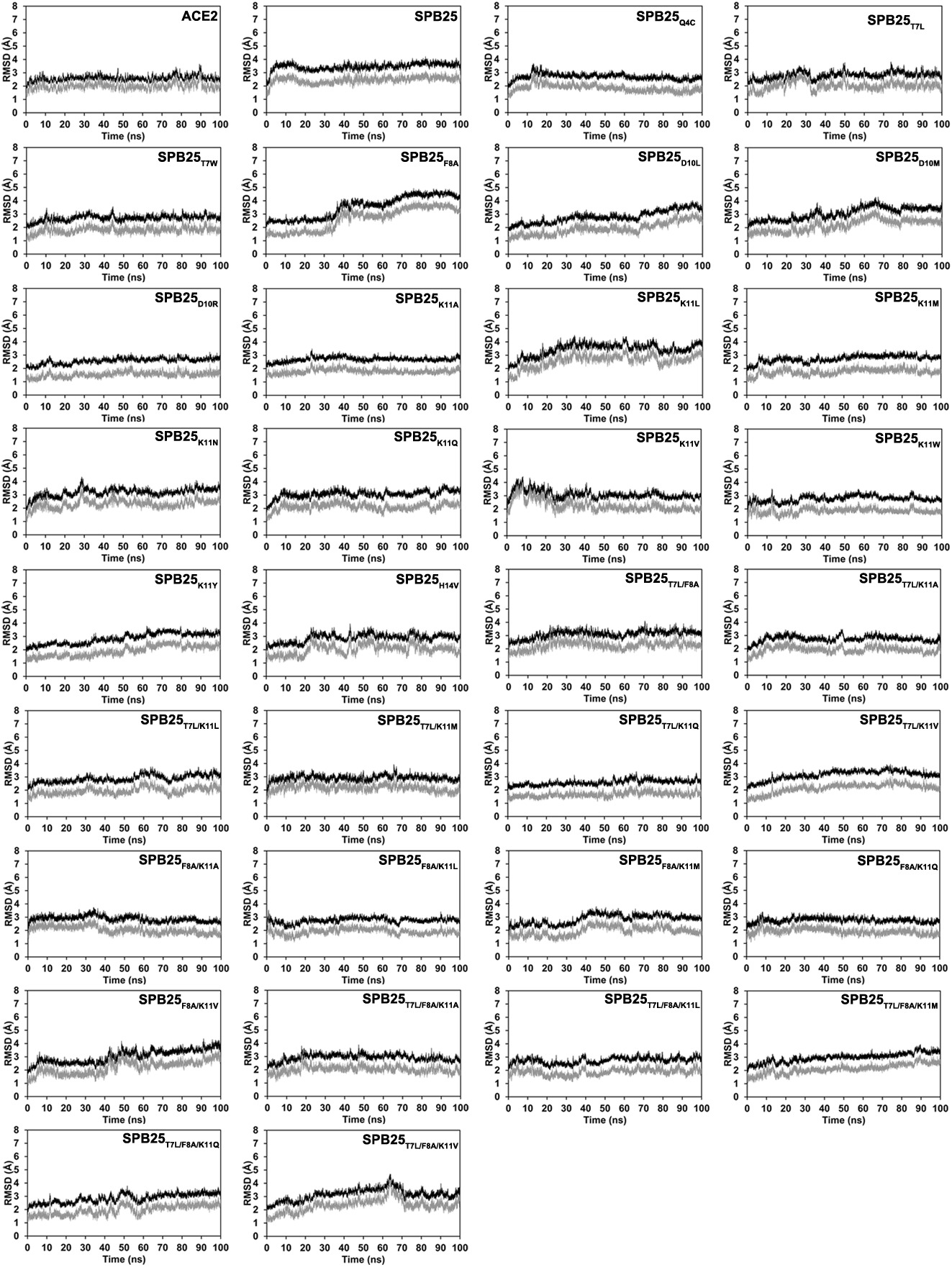

Supplement: S1 Fig — The RMSD values of all atoms and backbone atoms are shown in black and grey respectively. (TIF) [file pone.0292589.s001.tif]

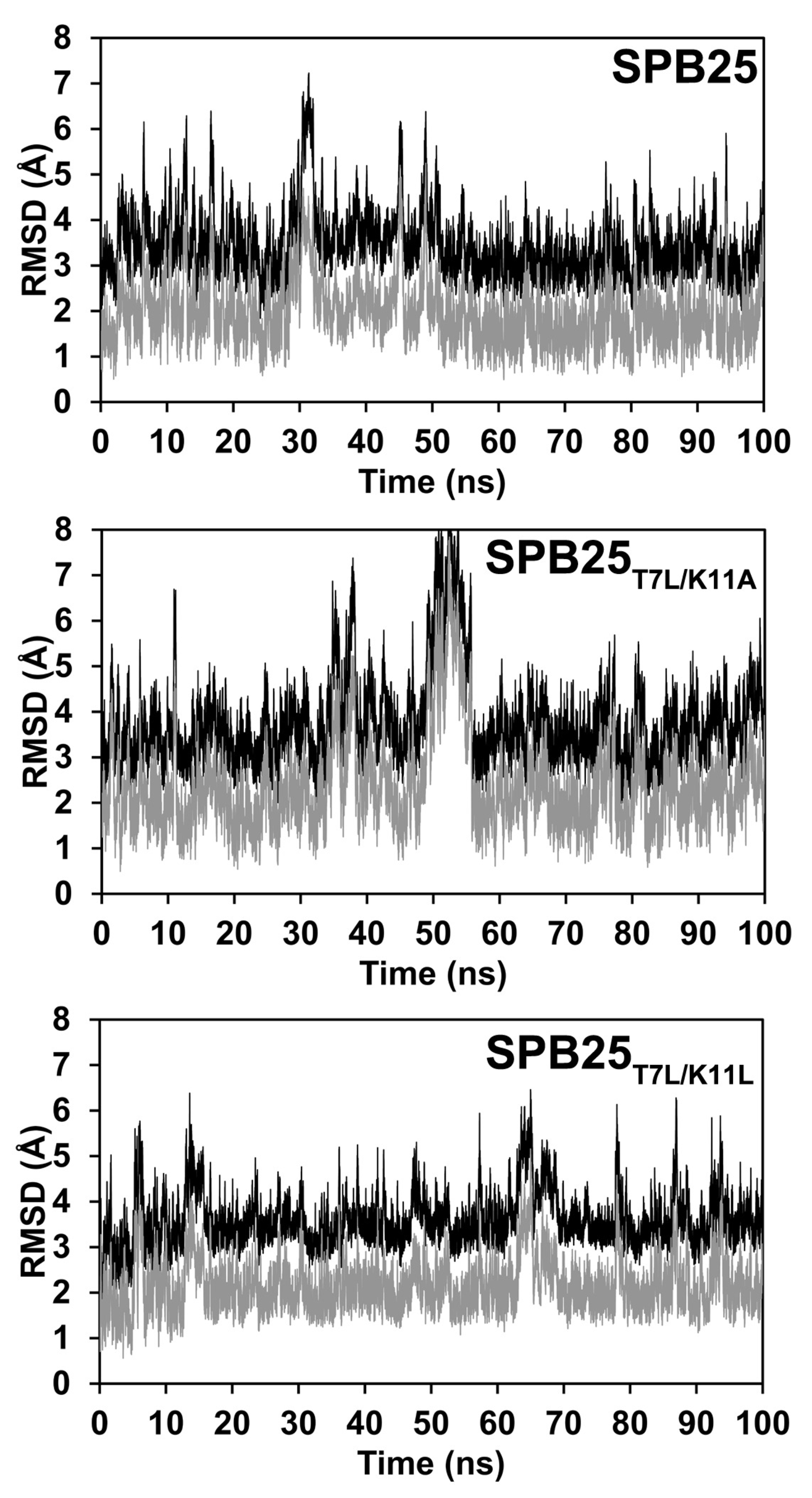

Supplement: S2 Fig — The RMSD values of all atoms and backbone atoms are shown in black and grey respectively. (TIF) [file pone.0292589.s002.tif]
