# Supplementary material for: Designing peptides predicted to bind to the omicron variant better than ACE2 via computational protein design and molecular dynamics
Source: PLoS One. 2023 Oct 10;18(10):e0292589. doi: 10.1371/journal.pone.0292589 (PMC10564162; doi:10.1371/journal.pone.0292589)
Supplement: S1 Table — (PDF) [file pone.0292589.s003.pdf]

S1 Table. The binding free energies of ACE2, SPB25 and all designed peptides to RBD of the omicron variant as calculated by Rosetta and MM-GBSA method.

System	$\Delta\Delta G_{bind\ (Rosetta)}$[a] (REU)	$\Delta G_{bind\ (MM\text{-}GBSA)}$ (kcal/mol)	$\Delta\Delta G_{bind\ (MM\text{-}GBSA)}$[b] (kcal/mol)
ACE2	-	−87.9 ± 0.5	−16.3 ± 0.7
SPB25	-	−71.6 ± 0.5	0.0 ± 0.7
$SPB25_{Q4A}$	1.7	-	-
$SPB25_{Q4C}$	−0.2	−65.7 ± 0.7	5.9 ± 0.9
$SPB25_{Q4E}$	0.1	-	-
$SPB25_{Q4I}$	0.2	-	-
$SPB25_{Q4S}$	3.3	-	-
$SPB25_{Q4V}$	1.4	-	-
$SPB25_{T7A}$	1.7	-	-
$SPB25_{T7H}$	2.2	-	-
$SPB25_{T7I}$	1.2	-	-
$SPB25_{T7L}$	−1.9	−74.7 ± 0.6	−3.1 ± 0.8
$SPB25_{T7N}$	2.5	-	-
$SPB25_{T7Q}$	0.4	-	-
$SPB25_{T7R}$	1.5	-	-
$SPB25_{T7W}$	−0.9	−61.6 ± 0.7	10.0 ± 0.9
$SPB25_{T7Y}$	1.0	-	-
$SPB25_{F8A}$	−0.2	−86.5 ± 0.6	−14.9 ± 0.8
$SPB25_{F8E}$	0.3	-	-
$SPB25_{F8K}$	0.9	-	-
$SPB25_{F8L}$	0.0	-	-
$SPB25_{F8R}$	0.9	-	-
$SPB25_{F8S}$	0.9	-	-
$SPB25_{F8T}$	1.6	-	-
$SPB25_{F8W}$	0.3	-	-
$SPB25_{F8Y}$	0.2	-	-
$SPB25_{D10A}$	0.0	-	-
$SPB25_{D10E}$	0.2	-	-
$SPB25_{D10H}$	0.6	-	-
$SPB25_{D10K}$	0.2	-	-
$SPB25_{D10L}$	−0.8	−70.1 ± 0.8	1.5 ± 0.9
$SPB25_{D10M}$	−1.7	−70.0 ± 0.7	1.6 ± 0.9
$SPB25_{D10N}$	0.8	-	-
$SPB25_{D10Q}$	0.4	-	-
$SPB25_{D10R}$	−2.4	−69.8 ± 0.5	1.8 ± 0.7
$SPB25_{D10S}$	1.3	-	-
$SPB25_{D10W}$	0.5	-	-
$SPB25_{D10Y}$	0.8	-	-
$SPB25_{K11A}$	−0.4	−73.2 ± 0.5	−1.6 ± 0.7
$SPB25_{K11C}$	0.4	-	-
$SPB25_{K11D}$	0.0	-	-
$SPB25_{K11E}$	2.6	-	-
$SPB25_{K11H}$	0.0	-	-
$SPB25_{K11L}$	−0.6	−80.8 ± 0.5	−9.2 ± 0.7
$SPB25_{K11M}$	−0.1	−79.4 ± 0.6	−7.8 ± 0.8
$SPB25_{K11N}$	−0.8	−66.2 ± 0.4	5.4 ± 0.6
$SPB25_{K11Q}$	−0.1	−77.4 ± 0.6	−5.8 ± 0.8

System	$\Delta\Delta G_{bind\ (Rosetta)}$[a] (REU)	$\Delta G_{bind\ (MM\text{-}GBSA)}$ (kcal/mol)	$\Delta\Delta G_{bind\ (MM\text{-}GBSA)}$[b] (kcal/mol)
$SPB25_{K11R}$	0.9	-	-
$SPB25_{K11S}$	2.0	-	-
$SPB25_{K11T}$	0.6	-	-
$SPB25_{K11V}$	−1.9	−81.2 ± 0.7	−9.6 ± 0.9
$SPB25_{K11W}$	−0.8	−61.3 ± 0.4	10.3 ± 0.6
$SPB25_{K11Y}$	−1.1	−61.9 ± 0.5	9.7 ± 0.7
$SPB25_{H14V}$	−0.7	−65.4 ± 0.5	6.2 ± 0.7
$SPB25_{T7L/F8A}$	−0.8	−84.6 ± 0.7	−13.0 ± 0.9
$SPB25_{T7L/K11A}$	0.9	−92.4 ± 0.4	−20.8 ± 0.6
$SPB25_{T7L/K11L}$	−2.1	−95.7 ± 0.5	−24.1 ± 0.7
$SPB25_{T7L/K11M}$	−0.1	−57.0 ± 0.7	14.6 ± 0.9
$SPB25_{T7L/K11Q}$	−1.1	−78.1 ± 0.5	−6.5 ± 0.7
$SPB25_{T7L/K11V}$	−1.9	−79.0 ± 0.6	−7.4 ± 0.8
$SPB25_{F8A/K11A}$	−0.1	−71.0 ± 0.4	0.6 ± 0.6
$SPB25_{F8A/K11L}$	−2.8	−73.7 ± 0.5	−2.1 ± 0.7
$SPB25_{F8A/K11M}$	−0.8	−74.7 ± 0.5	−3.1 ± 0.7
$SPB25_{F8A/K11Q}$	−2.0	−76.1 ± 0.6	−4.5 ± 0.8
$SPB25_{F8A/K11V}$	−2.1	−77.8 ± 0.7	−6.2 ± 0.9
$SPB25_{T7L/F8A/K11A}$	0.4	−51.6 ± 0.8	20.0 ± 0.9
$SPB25_{T7L/F8A/K11L}$	−3.1	−59.6 ± 0.4	12.0 ± 0.6
$SPB25_{T7L/F8A/K11M}$	−1.6	−59.1 ± 0.6	12.5 ± 0.8
$SPB25_{T7L/F8A/K11Q}$	−1.5	−76.8 ± 0.6	−5.2 ± 0.8
$SPB25_{T7L/F8A/K11V}$	−1.9	−67.0 ± 0.5	4.6 ± 0.7

[a] The difference between $\Delta G_{bind\ (Rosetta)}$ of a system and that of SPB25.

[b] The difference between $\Delta G_{bind\ (MM\text{-}GBSA)}$ of a system and that of SPB25.
