# Supplementary material for: Designing peptides predicted to bind to the omicron variant better than ACE2 via computational protein design and molecular dynamics
Source: PLoS One. 2023 Oct 10;18(10):e0292589. doi: 10.1371/journal.pone.0292589 (PMC10564162; doi:10.1371/journal.pone.0292589)
Supplement: S2 Table — (PDF) [file pone.0292589.s004.pdf]

S2 Table: Hydrogen bond occupations of ACE2, SPB25 and designed peptides binding to RBD of the omicron variant.

Acceptor[a]	DonorH[a]	Donor[a]	Hydrogen bond occupancy (%)			
			ACE2	SPB25	$SPB25_{T7L/K11A}$	$SPB25_{T7L/K11L}$
A475@O	S(19)@HG	S(19)@OG	51.2	-	-	-
A475@O	S(19)@H	S(19)@N	27.6	-	-	-
Q4(24)@OE1	N487@HD21	N487@ND2	61.8	-	-	-
R457@O	Q4(24)@HE21	Q4(24)@NE2	-	-	27.0	-
Q4(24)@OE1	Y473@HH	Y473@OH	-	0.1	-	45.8
T/L7(27)@OG1	Y473@HH	Y473@OH	-	70.5	-	-
D10(30)@OD1	N417@HD21	N417@ND2	-	95.7	11.5	0.7
D10(30)@OD2	Y421@HH	Y421@OH	-	58.3	-	-
D10(30)@OD1	R403@HH22	R403@NH2	-	-	74.0	-
D10(30)@OD2	R403@HH22	R403@NH2	-	-	73.0	-
D10(30)@OD2	R403@HH12	R403@NH1	-	-	42.4	-
D10(30)@OD1	R403@HH12	R403@NH1	-	-	31.4	-
D10(30)@OD2	Y453@HH	Y453@OH	-	-	35.9	8.5
D10(30)@OD1	Y453@HH	Y453@OH	-	-	27.6	9.3
H14(34)@ND1	R493@HE	R493@NE	98.8	-	86.1	88.7
H14(34)@ND1	R493@HH21	R493@NH2	96.5	-	98.3	99.7
S494@O	H14(34)@HE2	H14(34)@NE2	34.3	-	-	-
H14(34)@ND1	R493@HH11	R493@NH1	-	98.0	-	-
Y501@OH	H14(34)@HE2	H14(34)@NE2	-	-	93.1	0.2
E15(35)@OE1	R493@HH22	R493@NH2	59.3	-	34.4	42.0
E15(35)@OE2	R493@HH12	R493@NH1	49.5	-	60.8	47.9
E15(35)@OE2	R493@HH22	R493@NH2	60.2	-	59.0	56.9
E15(35)@OE1	R493@HH12	R493@NH1	47.8	-	36.2	38.6
E17(37)@OE1	R403@HH12	R403@NH1	-	91.0	-	6.1
E17(37)@OE2	R403@HH12	R403@NH1	-	91.6	-	6.3
E17(37)@OE2	R403@HH22	R403@NH2	-	44.7	-	10.6
E17(37)@OE1	R403@HH22	R403@NH2	-	56.0	-	10.7
D18(38)@OD1	Y449@HH	Y449@OH	69.2	-	-	-
D18(38)@OD2	Y449@HH	Y449@OH	55.8	-	6.7	-
D18(38)@OD1	R493@HH12	R493@NH1	-	26.3	-	-
D18(38)@OD2	R493@HH12	R493@NH1	-	76.8	-	-
D18(38)@OD2	R493@HH22	R493@NH2	-	75.9	-	-
D18(38)@OD1	R498@HE	R498@NE	-	-	99.9	6.8
D18(38)@OD2	R498@HH21	R498@NH2	0.7	-	98.6	78.2
D18(38)@OD1	R498@HH21	R498@NH2	-	-	75.1	91.7
D18(38)@OD1	Y501@HH	Y501@OH	-	-	99.9	1.2

Acceptor[a]	DonorH[a]	Donor[a]	Hydrogen bond occupancy (%)			
			ACE2	SPB25	$SPB25_{T7L/K11A}$	$SPB25_{T7L/K11L}$
D18(38)@OD2	R498@HE	R498@NE	-	-	14.0	93.6
D18(38)@OD2	Y501@HH	Y501@OH	-	-	0.4	99.7
Y21(41)@OH	T500@HG1	T500@OG1	40.9	-	8.4	-
Y21(41)@OH	G502@H	G502@N	-	-	25.3	33.2
Y21(41)@O	T500@HG1	T500@OG1	-	-	28.2	35.5
N487@OD1	Y(83)@HH	Y(83)@OH	97.6	-	-	-
K353@O	G502@H	G502@N	99.8	-	-	-
D(355)@OD2	T500@HG1	T500@OG1	74.2	-	-	-

[a] The residue number of ACE2 is in parenthesis.
